# Muscle Network Connectivity Study in Diabetic Peripheral Neuropathy Patients

**DOI:** 10.3390/s24154954

**Published:** 2024-07-31

**Authors:** Isabel Junquera-Godoy, José Luís Martinez-De-Juan, Gemma González-Lorente, José Miguel Carot-Sierra, Julio Gomis-Tena, Javier Saiz, Silvia García-Blasco, Isabel Pertusa-Mazón, Esther Soler-Climent, Gema Prats-Boluda

**Affiliations:** 1 Centro de Investigación e Innovación en Bioingeniería (Ci2B), Universitat Politècnica de València (UPV), 46022 Valencia, Spain; ijungod@ci2b.upv.es (I.J.-G.); ggonlor@etsii.upv.es (G.G.-L.); jgomiste@ci2b.upv.es (J.G.-T.); jsaiz@ci2b.upv.es (J.S.); gprats@ci2b.upv.es (G.P.-B.); 2 Departamento de Estadística e Investigación Operativa Aplicadas y Calidad, Universitat Politècnica de València (UPV), 46022 Valencia, Spain; jcarot@eio.upv.es; 3 Servicio de Rehabilitación, Departamento Salud Elche Hospital General de FISABIO, 03203 Elche, Spain; silvia.garciab@umh.es (S.G.-B.); pertusa_isa@gva.es (I.P.-M.); 4 Área de Investigación en Enfermería-Fisioterapia, Departamento Salud Elche Hospital General de FISABIO, 03203 Elche, Spain; soler_estcli@gva.es

**Keywords:** electromyography, diabetic peripheral neuropathy, biomarker, muscle interaction, transfer entropy

## Abstract

Diabetic peripheral neuropathy (DPN) is a prevalent complication of chronic diabetes mellitus and has a significant impact on quality of life. DPN typically manifests itself as a symmetrical, length-dependent sensorimotor polyneuropathy with severe effects on gait. Surface electromyography (sEMG) is a valuable low-cost tool for assessing muscle activation patterns and precise identification of abnormalities. For the present study, we used information theory methods, such as cross-correlation (CC), normalized mutual information (NMI), conditional granger causality (CG-Causality), and transfer entropy (TE), to evaluate muscle network connectivity in three population groups: 33 controls (healthy volunteers, CT), 10 diabetic patients with a low risk of DPN (LW), and 17 moderate/high risk patients (MH). The results obtained indicated significant alterations in the intermuscular coupling mechanisms due to diabetes and DPN, with the TE group showing the best performance in detecting differences. The data revealed a significant increase in information transfer and muscle connectivity in the LW group over the CT group, while the MH group obtained significantly lower values for these metrics than the other two groups. These findings highlight the sEMG coupling metrics’ potential to reveal neuromuscular mechanisms that could aid the development of targeted rehabilitation strategies and help monitor DPN patients.

## 1. Introduction

Diabetic peripheral neuropathy (DPN) is a prevalent and burdensome complication of chronic diabetes mellitus, characterized by pain, paresthesia, and sensory loss. It affects around 50% of individuals with diabetes, significantly influences their quality of life, and leads to considerable morbidity and mortality [1]. Diabetes is now reaching epidemic proportions, and the global patient population is anticipated to double by 2030, with over 366 million individuals, according to the International Diabetes Federation [2]. The most common complication of diabetes, DPN, has a large impact on healthcare costs. The total yearly per-patient cost averages $6632, with a twofold increase in individuals with peripheral neuropathy and a fourfold rise in those experiencing moderate to severe pain [3]. This financial strain is further exacerbated by higher healthcare use associated with DPN-related complications, such as foot ulcers and amputations [4].

DPN typically appears as symmetrical, length-dependent sensorimotor polyneuropathy, starting in the toes and gradually progressing to the upper limbs [5]. It primarily affects the intrinsic muscles of the foot, including the extensor digitorum brevis and flexor digitorum brevis, the lower leg muscles, such as the tibialis anterior, and the intrinsic muscles of the hand [6]. Painful symptoms, including burning and tingling sensations, are present in approximately one-third of DPN patients and can severely disrupt their sleep and daily activities [7]. Despite extensive research, DPN pathogenesis remains multifactorial and complex, involving metabolic, vascular, and inflammatory mechanisms. Treatment is challenging, with the limited efficiency of the available pharmacotherapies and a need for improved management strategies [4]. There is also a growing recognition of the impact of non-pharmacological interventions, emphasizing the importance of holistic approaches in addressing this debilitating condition. As the prevalence of diabetes continues to rise, there is an urgent need for further research and development of effective therapies to alleviate the considerable DPN burden on individuals and healthcare systems worldwide [8].

DPN significantly affects the patients’ gait in a range of deficits that compromise mobility and independence. These include reduced walking speed, shortened stride length, and impaired balance, all of which are critical for everyday functioning [9]. The primary cause of these abnormalities is damaged sensory and motor nerves due to chronic hyperglycemia, which leads to diminished proprioception and muscle strength [10]. Proprioception is the sense of self-movement and body position and is crucial for coordinating movements and maintaining balance. The loss of proprioceptive feedback in DPN results in an inability to correctly sense foot position when walking, leading to unsteady and abnormal gait patterns and reduced muscle strength [11]. Motor unit (MU) loss leads to muscle weakness and is closely related to atrophy and fat infiltration [6]. Abnormal muscle activation patterns, such as delayed muscle activation, increased coactivation, or prolonged muscle activity, further contribute to inefficient and unstable gait, increasing the risk of falls and significantly affecting quality of life [12,13].

Physical therapy is a cornerstone intervention in addressing gait abnormalities, involving therapeutic exercises designed to improve muscle strength and coordination to restore normal gait patterns. Balance training exercises help patients develop better stability and prevent falls. Resistance exercises aim to strengthen specific muscle groups, particularly those in the lower limbs associated with DPN [6]. However, the effectiveness of these therapeutic measures greatly depends on the ability to monitor the patients’ progress and adapt treatment plans as required. An objective assessment method is crucial to accurately gauge the results, track the patients’ improvement over time, and refine the therapeutic approaches to ensure optimal patient outcomes [6].

Surface electromyography (sEMG) is a valuable low-cost tool for understanding and assessing muscle activation patterns by non-invasively measuring electrical muscle activity [14] and could reveal how neuropathy affects muscle coordination and its contribution to abnormal activation patterns in DPN patients. By identifying these deficits, clinicians could design individual treatments to correct specific muscle activation issues. Regular sEMG assessment can help monitor changes in muscle activity over time, making it an essential tool for evaluating the effectiveness of the therapy.

Recent advances in sEMG research have revealed how neuropathy alters leg muscle activation. Studies have shown that these patients often have increased co-activation of antagonistic muscles during voluntary contractions in addition to reduced contractile properties in the ankle dorsiflexors [12]. Other studies have reported a delayed onset of muscle activity in key muscles such as the quadriceps and gastrocnemius, which can impair muscle contraction timing for stable walking [13]. However, in [15], the early activation of the lower limb muscles during ascending and descending steps and in [16], during gait, was used to compensate for the delay in MU recruitment. 

Determining the complex neuromuscular control mechanisms in DPN extends beyond individual muscle activity; it also entails quantifying intermuscular interactions to decipher muscle coupling and coordination patterns. This requires sophisticated analytical tools such as information theory methods such as mutual information (MI), cross-correlation (CC), granger causality (GC), and transfer entropy (TE). These methods offer model-free approaches that capture both linear and non-linear relationships, making them effective in quantifying functional neurophysiological coupling in DPN [17,18,19]. Although many studies have used sEMG to detect muscle alterations, most focused on individual muscle assessments [13,15,20,21]. So far, no studies have used advanced information theory metrics to analyze the possible changes in muscle network connectivity due to DPN. These innovative metrics have been reported in other contexts, including Parkinson’s disease, stroke, exoskeleton-assisted movement, and mapping postural control in healthy individuals [22,23,24,25]. Applying these methods to DPN research could provide significant insights into muscle coordination deficits and network alterations.

The primary objective of this study was thus to comprehensively evaluate and compare variations in muscle network connectivity and intermuscular coupling among individuals diagnosed with diabetes and DPN by means of information theory methods. The goal was to discern any muscle activation and intermuscular coupling patterns that could be used as reliable and objective biomarkers to assess DPN severity and progress and to evaluate the effectiveness of therapeutic interventions.

## 2. Materials and Methods

### 2.1. Participants

A total of 60 participants (age: 56 ± 14 years; 25 males, 35 females) volunteered for the study, of whom 27 had been diagnosed with diabetes mellitus, while the remainder comprised the control group. The subjects in the diabetic cohort underwent foot risk assessments and were categorized into low, moderate, or high-risk groups. Foot ulcers or amputations were exclusion criteria. The diabetic subgroup comprised 10 low, 6 moderate, and 11 high-risk individuals. Three distinct population groups were established: controls (CT), diabetics with a low risk of diabetic peripheral neuropathy (LW), and those with a moderate/high risk (MH), the latter indicating active peripheral neuropathy. On the other hand, despite not showing apparent signs, the low-risk group could still have been either in the initial stages of the condition or have asymptomatic diabetic peripheral neuropathy [26]. The research protocol received the approval of the Hospital General Universitario de Elche (PI 138/2022) and the Ethics Committee of the Universitat Politècnica de València (P04_27_01_202) and adhered to the principles of the Declaration of Helsinki. Before participating, the subjects were informed of the nature of the study and required to sign written consent forms.

### 2.2. Experimental Protocol and Data Acquisition

The experiment consisted of a dynamic exercise of continuous alternated dorsiflexion and plantar flexion movements until claudication. Dorsiflexion involved the participant decreasing the angle between the dorsum (top) of the foot and the anterior (front) part of the leg by pulling the toes and the top of the foot upwards towards the shin as far as possible without causing discomfort, typically achieving a range of motion of approximately 20 degrees from the neutral position. Conversely, plantar flexion required the participant to increase the angle between the dorsum of the foot and the anterior part of the leg by pointing the toes and the top of the foot downwards away from the shin, with a normal range of motion of approximately 50 degrees from the neutral position. These movements were performed continuously at a steady pace of approximately one second per movement until the participant experienced claudication. The participants carried out two repetitions of the exercise, separated by a 30 s interval. Each repetition of the exercise lasted approximately 120 s, with a maximum of 150. Bipolar surface EMG was recorded by a Noraxon Ultium EMG sensor (Scottsdale, AZ, USA) from 4 muscles in the lower right leg and foot simultaneously: the tibialis anterior (TA), medial gastrocnemius (GM), extensor digitorum brevis (ED), and flexor digitorum brevis (FD). The Ag/AgCl surface electrode placements were based on SENIAM guidelines [27]. TA and ED muscles co-activated during dorsiflexion, while the GM and FD muscles co-activated during the dynamic plantar flexion exercise. The analyzed connectivity of the muscle network was therefore composed of 4 muscles (TA, GM, ED, and FD), resulting in 6 different muscle pairs (TA-GM, TA-ED, TA-FD, GM-ED, GM-FD, and ED-FD).

sEMG signals were sampled at 2 kHz for data acquisition and analyzed on MATLAB software (R2022b, MathWorks, Natick, MA, USA). Prior to the muscle connectivity analysis, the signals were filtered with a 4th-order zero-phase band-pass Butterworth filter with a frequency band of 20–500 Hz. The filtered sEMG data was divided into W non-overlapping windows, with each window containing N = 12,000 samples. Within each window, four information theory parameters were calculated. Since there were no significant statistical differences between the two dynamic exercises, the average values of each parameter were computed across all windows from both exercises for each participant.

**Figure 1:**
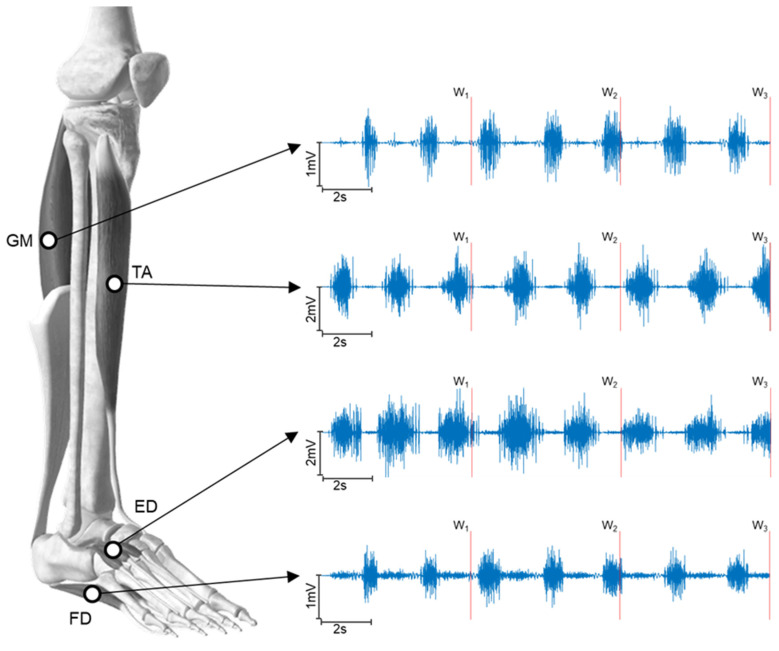
Temporal representation of the raw sEMG signal from a participant (CT group). From top to bottom, medial gastrocnemius (GM), tibialis anterior (TA), extensor digitorum brevis (ED), and flexor digitorum brevis (FD) muscles are divided into three 6 s windows (W1, W2, W3) during an 18 s segment of a dynamic exercise.

Figure 1 shows an example of an 18 s segment from the four raw sEMG signals simultaneously recorded during a dynamic exercise. The 6 s windows into which the signals were subdivided are also marked by a vertical red line. As can be seen, the TA and ED muscle activations occurred simultaneously during the dorsiflexion phase, while the GM and FD muscles coactivated during the plantar flexion phase of the dynamic exercise.

### 2.3. Information Theory Parameters

The following 4 parameters were computed to identify changes in the muscular connectivity network between the population groups: cross-correlation (CC), normalized mutual information (NMI), conditional granger causality (CG-Causality) and transfer entropy (TE).

#### 2.3.1. Cross-Correlation

The maximum of the cross-correlation (CC) between two time series data sets represents the highest similarity or alignment between the two series when one is shifted relative to the other. Considering two time series $x_{n}$ and $y_{n}$ where n=0, 1, 2, …, N−1, the cross-correlation is given by:

(1) $$R_{xy}\left(m\right)=\left\{\begin{array}{c} \sum_{n=0}^{N-m-1}x_{n+m}\;y_{n}^{*},\;\;\;m\geq 0,\\ R_{xy}^{*}\left(-m\right),m<0. \end{array}\right.\;\;$$

(2) $$c(m)=R_{xy}(n-N),\;\;m=1,2,...,\;2N-1.\;$$

(3) $$CC=max\left(c\left(m\right)\right)\;$$

where $R_{xy}(m)$ is the cross-correlation function, *m* represents the lag or time shift, c(m) the cross-correlation sequence, and CC the highest degree of correlation or synchronization. CC is a scalar between 0 and 1 which two curves with the same shape will have a CC=1. The maximum cross-correlation between pairs of preprocessed sEMG curves was performed as in [28].

#### 2.3.2. Normalized Mutual Information

Mutual information (MI) quantifies the information shared between two variables or time series data (X and Y) and can be defined as a function of Shannon entropies and conditional entropies [17]:

(4) $$I_{XY}=H_{X}-H_{X|Y}=H_{Y}-H_{Y|X}\;\;$$

where $I_{XY}$ is the MI between variables X and Y, $H_{X}$ and $H_{Y}$ are the Shannon entropies of *X* and *Y* respectively. Moreover, $H_{X|Y}$ and $H_{Y|X}$ are the conditional entropies of *X* given *Y* and *Y* given *X* respectively.

(5) $$I_{XY}=\sum_{x_{i}y_{i}}P_{XY}(x_{i},\;y_{i})\log\frac{P_{XY}(x_{i},y_{i})}{P_{X}(x_{i})P_{Y}(y_{i})}$$

where $P_{XY}$ is the joint probability mass function of X and Y and $P_{X}$ and $P_{Y}$ are the marginal probability mass functions of X and Y respectively. Normalizing the MI by the minimum of the entropies as:

(6) $${NMI}_{XY}=\frac{I_{XY}}{min(H_{X},H_{Y})}$$

The entropies can be defined in terms of probability of distribution as:

(7) $$H_{X}=-\sum_{x_{i}}P_{X}(x_{i})\log\left(P_{X}(x_{i})\right)$$

(8) $$H_{Y}=-\sum_{y_{i}}P_{Y}(y_{i})\log\left(P_{Y}(y_{i})\right)$$

where ${NMI}_{XY}$ is the normalized MI between the variables X and Y. NMI is a scalar between 0 and 1, being 0 when the time series are independent. The higher the correlation between the pair of EMG signals, the higher the value of the MI, and the higher the muscle coupling. The behavior of this parameter is expected to be comparable to the *CC* but with a non-linear approach.

#### 2.3.3. Conditional Granger Causality

Granger Causality (GC) analysis was computed to measure the predictive power of past signal *Y* on signal *X*. This statistical measure determines whether the past values of *Y* provide additional information in predicting the future values of *X* beyond what is already explained by *X*’s own past [19]. 

Mathematically, this process involves the creation and comparison of two linear vector autoregressive models (VAR). The first model is described as a linear combination of its own past values and those of *Y*, expressed by the following equation:

(9) $$X\left(t\right)=\sum_{k=1}^{p}A_{xx,k}.X_{t-k}+\sum_{k=1}^{p}A_{xy,k}.Y_{t-k}+\varepsilon_{x,t}\;$$

where *p* is the model order, $A_{xx,k}$ and $A_{xy,k}$ are the regression coefficients, and $\varepsilon_{x,t}$ is the error. The second model is the reduced model, defined only by X past values to predict X current values.

(10) $$X(t)=\sum_{k=1}^{p}{A^{\prime }}_{xx,k}.X_{t-k}+{\varepsilon^{\prime }}_{x,t}\;$$

By taking the logarithmic ratio between the covariance of the two residuals, we can get the estimate *GC* describing *Y* as the G-causal of X.

(11) $${GC}_{Y\to X}=\ln\frac{Cov({\varepsilon^{\prime }}_{x,t})}{Cov(\varepsilon_{x,t})}\;$$

A limitation of unconditional *GC* lies in the potential for spurious causal relationships to emerge when a third variable Z influences both X and Y. In contrast, conditional Granger causality (CG-Causality) addresses this issue by considering the interdependent nature of multivariate time series data. It achieves this by incorporating the variable *Z* into both the reduced and full regression models, thereby mitigating the risk of misleading causal inferences.

(12) $${CG-Causality}_{Y\to X|Z}=\ln\frac{Cov({\varepsilon^{\prime }}_{x,t})}{Cov(\varepsilon_{x,t})}$$

Analyzing EMG signals, CG-Causality enabled the detection of linear interactions between muscles and unusual causal influences. This parameter gives directionality, revealing the extent to which DPN influences the causal relationship between muscles, potentially designating one muscle as the “director” of another, contrasting their relationship in the absence of neuropathy.

CG-Causality was operationalized by a multivariate, model-based approach using the VAR model theory [29]. This approach involved applying the CG-Causality on windows containing 12,000 samples. The information criteria Akaike with a maximum model order of 20 was used for a model order estimation. 

The resulting regression coefficients were used to derive autocovariance sequences, leading to the computation of pairwise-conditional time-domain Multivariate Granger Causalities (MVGCs) [30].

#### 2.3.4. Transfer Entropy

We also computed the transfer entropy (TE) between time series *X* and *Y* with the flow of information directed from *Y* to *X*. The probability density estimation is based on the Darbellay-Vajda (D-V) partitioning algorithm [31]. *TE* quantifies the amount of information transferred from one variable to another. It is non-parametric and capable of capturing non-linear coupling effects, making it valuable for analyzing complex systems with minimal prior knowledge. Unlike mutual information, transfer entropy is asymmetric, conveying directional information and serving as a non-linear extension of GC [18].

**Figure 2:**
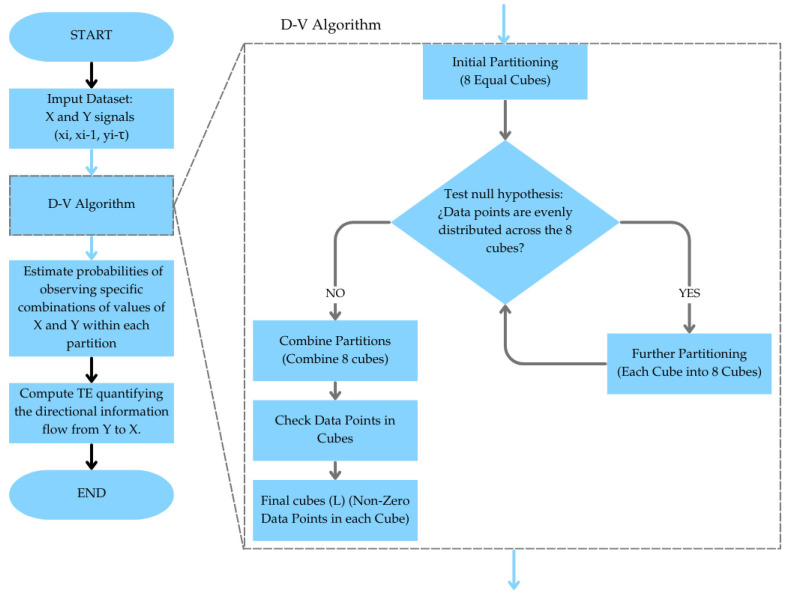
Flowchart illustrating the process of estimating transfer entropy (TE) detailing the steps of the D-V algorithm.

Estimating *TE* with adaptive partitioning using the D-V algorithm involves several key steps, as shown in Figure 2. First, the joint space of the two time series data *X* and *Y* is partitioned into smaller regions or partitions. This adaptive partitioning is achieved through the D-V algorithm, which adjusts partition boundaries based on the local density of data points to capture intricate dependencies between *X* and *Y* [18]. The probabilities of observing specific combinations of values of *X* and *Y* within each partition are then estimated, accounting for the dynamic nature of the data distribution. Finally, *TE*, quantifying the directional information flow from *Y* to *X*, is computed based on these probabilities. This approach offers a robust and flexible method of assessing information transfer and directional relationships between variables in dynamic systems, leveraging adaptive partitioning to accurately capture complex data dynamics. The D-V algorithm recursively partitions the three-dimensional space defined by $x_{i},\;x_{i-1}$, and $y_{i-\tau }$ into cubes of varying sizes. Mathematically, this process can be described by the following equation:

(13) $${TE}_{Y\to X}(\tau )\sim \sum_{k=1}^{L}\frac{n_{k}}{P}\log\frac{n_{k}n_{k}^{x_{i-1}}}{n_{k}^{x_{i-1},y_{i-\tau }}n_{k}^{x_{i},x_{i-1}}}$$

where L is the finite number of cubes, P is the total number of triplets ($x_{i},\;x_{i-1},$ and $y_{i-\tau }$), $n_{k}$ is the number of data points in the *k*th partition, and $n_{k}^{x_{i-1}}$, $n_{k}^{x_{i},x_{i-1}}$ and $n_{k}^{x_{i-1},y_{i-\tau }}$ are the numbers of data points in the entire data that are within the bounds of the *k*th partition.

#### 2.3.5. Statistical Analysis

An initial analysis was performed using the analysis of variance (ANOVA) test to quantify differences in muscle connectivity between the various population groups, after which a more in-depth examination of group differences was conducted using the least significant difference (LSD) test to assess all pairwise comparisons. A parametric paired *t*-test was also performed within each group population to determine the significance of this directionality in the parameters with directionality (CG-Causality and TE). The threshold of significance of the statistical analyses was set to *p* < 0.05.

## 3. Results

Following the method outlined above, parameters were computed to capture the interactions between the TA, GM, ED, and FD muscles. To initially assess whether there were any differences between the groups, an ANOVA test was performed. This test provided a general overview of differences without identifying which specific groups differed from each other. For a more detailed examination of specific group differences, pairwise comparisons were conducted using the LSD test. The resulting *p*-values are given in Table 1, Table 2 and Table 3. Table 1 gives the results of the ANOVA test, highlighting general differences for the four parameters between the groups. For the pairwise comparisons using the LSD test, Table 2 categorizes the parameters without directionality (CC and NMI), while Table 3 organizes the parameters with directionality (GC-Causality and TE) based on the direction of the interactions. To enhance the clarity of our findings, we have focused on the discussion of the statistically significant results (*p* < 0.05).

The ANOVA results in Table 1 show that the TA-ED muscle pair exhibits statistically significant differences (*p* < 0.05) between groups across all the parameters, while NMI shows significant differences between groups for the GM-FD pair. Significant differences can also be seen for the GM-FD pair in the TE parameter for the GM → FD direction, with near-threshold *p*-values in the opposite direction and in the GC-Causality parameter for both directions. The TA-GM, TA-FD, and ED-FD muscle pairs do not present statistically significant differences in any parameter in this first evaluation of group differences. To further identify between which groups these differences appear, the pairwise comparison using the LSD test was computed.

The pairwise comparison results from the LSD test (Table 2) indicate significant differences (*p* < 0.05) in the CC parameter for the TA-GM muscle pair between the CT and MH groups. For the TA-ED muscle pair, significant differences can be seen between CT and MH, and between LW and MH. As in the CC, the NMI parameter also presents significant differences for the TA-ED muscle pair between CT and MH and between LW and MH, the GM-FD pair shows a significant difference between CT and LW. 

As can be seen in Table 3, the CG-Causality parameter is significantly different in the TA-ED and GM-FD muscle pairs in both directions for comparisons involving MH (CT-MH and LW-MH). Near-threshold differences also occurred in the TA → GM direction between CT and LW and in GM-ED (both directions) between CT and MH, as well as in GM → FD between LW and MH. For the TE parameter, significant differences are found in the TA-ED pair between CT and MH and between LW and MH in both directions. The LW and MH comparison also presents differences in the TA → GM and GM → FD pairs. Comparing CT and LW, statistically significant differences are found in the GM-FD (both directions), GM → ED and ED ← FD pairs. This group comparison would allow us to identify changes in the LW group and facilitate the early detection of DPN. 

**Figure 3:**
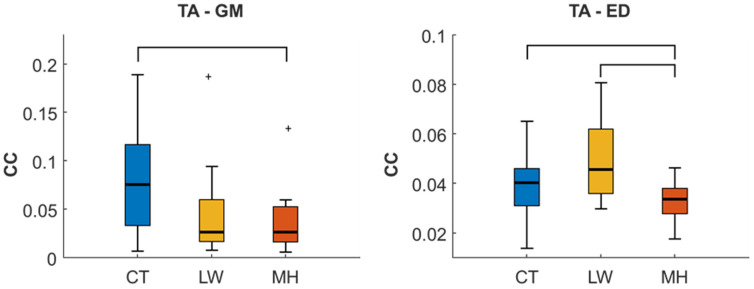
Box and Whisker plots of the CC parameter of TA-GM and TA-ED muscle pairs from the CT, LW, and MH populations. Statistically significant differences (*p* < 0.05) between the LSD test groups are shown in the black brackets. Outlier values are shown as ‘+’.

**Figure 4:**
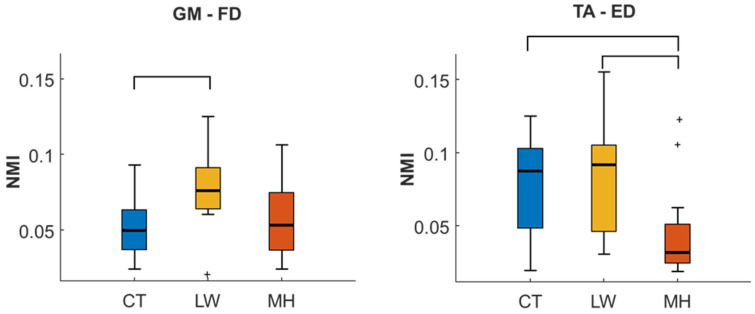
Box and Whisker plots for NMI of TA-ED and GM-FD muscle pairs from the CT, LW, and MH muscle groups. Statistically significant differences (*p* < 0.05) between groups from the LSD test are given in black brackets. Outlier values are shown as ‘+’.

**Figure 5:**
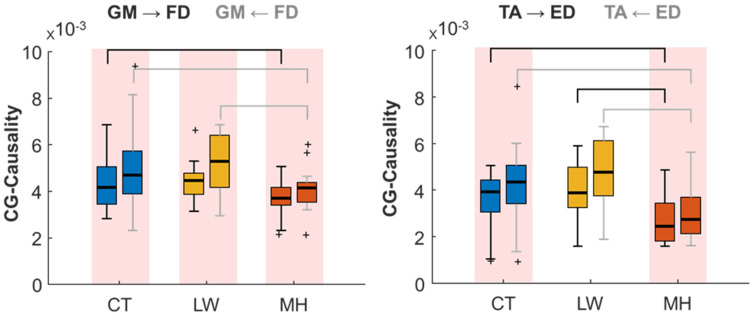
Box and Whisker plots depicting the CG-Causality distribution across TA-ED and GM-FD muscle pairs for CT, LW, and MH population groups in bidirectional comparison. Whiskers in black denote one directionality, while grey indicates the opposite. Statistically significant differences (*p* < 0.05) between groups, as determined by the LSD test, are denoted by brackets in both black and grey. Statistically significant differences (*p* < 0.05) between directionalities, as determined by the *t*-test, are denoted by a red background. Outlier values are shown as ‘+’.

**Figure 6:**
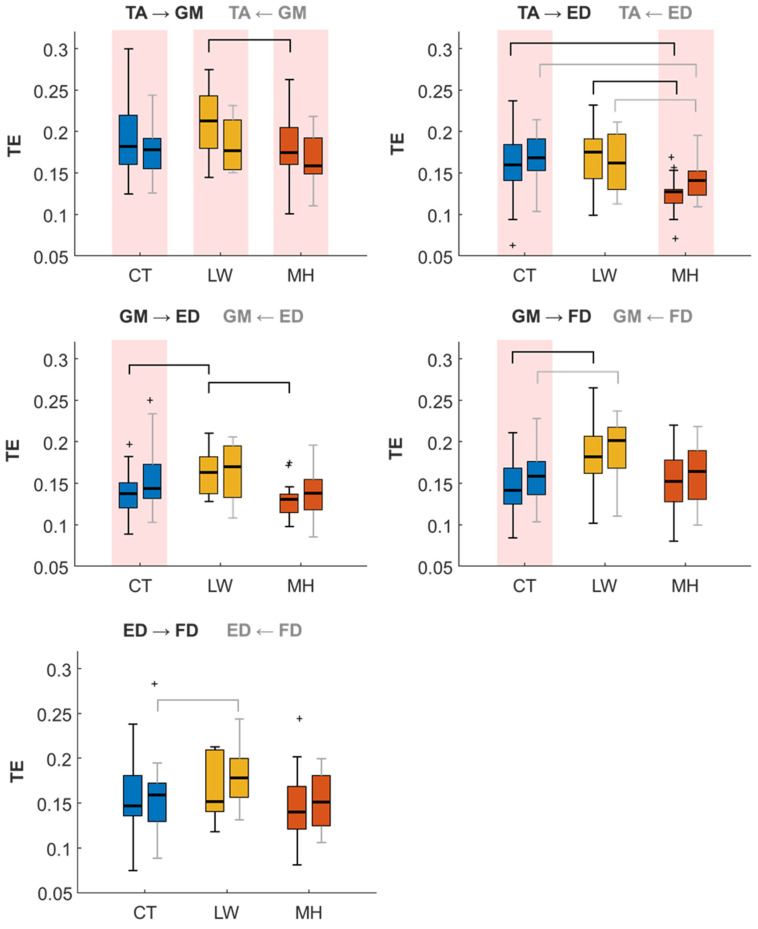
Box and Whisker plots depicting the TE parameter across TA-GM, TA-ED, GM-ED, GM-FD, and ED-FD muscle pairs for three CT, LW, and MH groups in bidirectional comparison. Whiskers in black denote one directionality, while grey indicates the opposite. Statistically significant differences (*p* < 0.05) between groups, as determined by the LSD test, are denoted by both black and grey brackets. Statistically significant differences (*p* < 0.05) between directionalities, as determined by the *t*-test, are denoted by a red background. Outlier values are shown as ‘+’.

**Figure 7:**
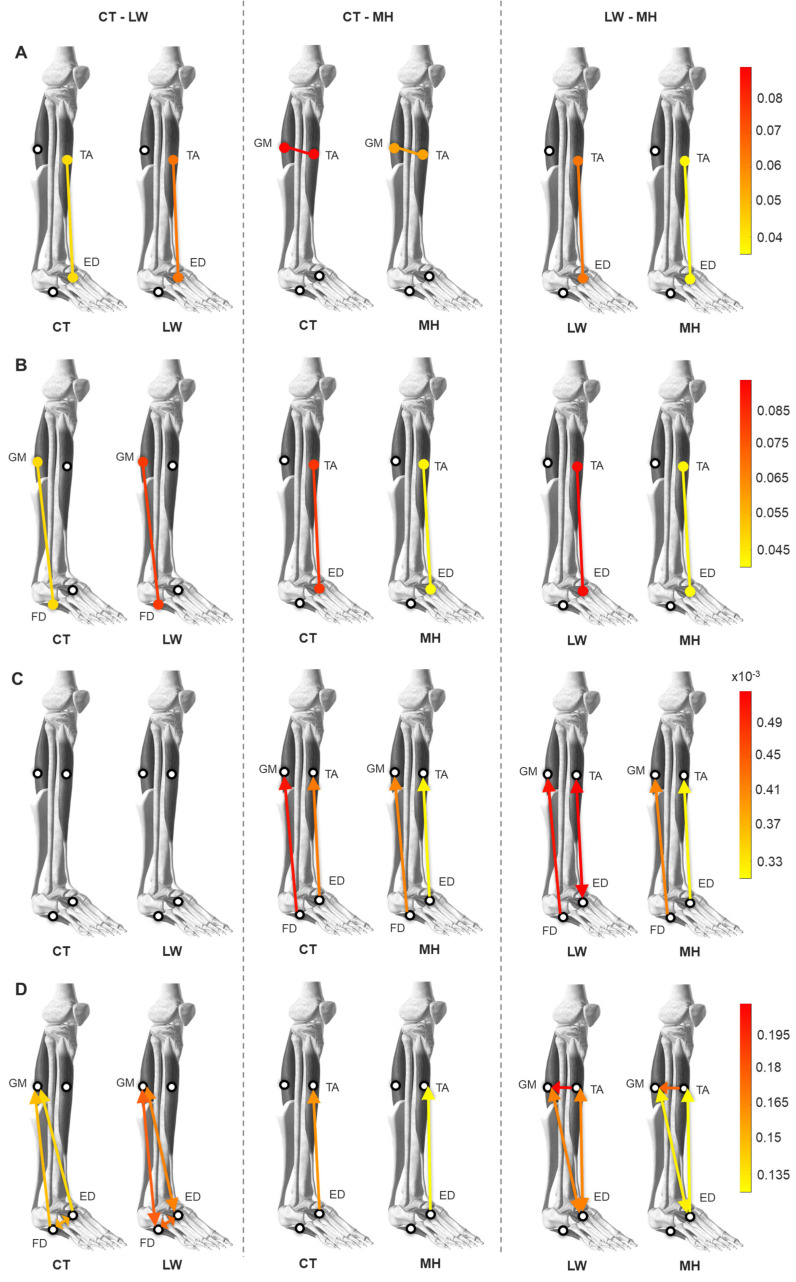
Visual representation of significant muscle interactions between the muscles medial gastrocnemius (GM), tibialis anterior (TA), extensor digitorum brevis (ED), and flexor digitorum brevis (FD) observed from the (**A**) CC, (**B**) NMI, (**C**) CG-Causality, and (**D**) TE parameters when comparing the CT-LW, CT-MH, and LW-MH populations. The color of the lines represents the mean parameter of each group in that specific muscle pair, while the arrow represents its statistically predominant directionality. The bidirectional arrow represents non-significant directionality.

Figure 3, Figure 4, Figure 5, Figure 6 and Figure 7 show the distribution of the values for the CC, NMI, CG-Causality, and TE parameters from the muscle pairs with statistically significant differences between the groups. Figure 3 gives the distribution of the CC parameter values for the TA-GM and TA-ED muscle interactions, which were significantly different between the groups. For the TA-GM pair, there is a reduction of the CC in the diabetic cohort; the difference between CT and MH is statistically significant. In TA-ED, the LWs showed higher values than those of CT and MH, with statistical significance for this second group, while the values for MH were significantly lower than CT.

Figure 4 gives the distribution of the NMI values for the two muscle pairs with significant differences between the groups. The TA-ED pair presents very similar results to those obtained from the CC parameter, with a reduction of the NMI for the MH group and statistically significant differences from the other two groups. However, while the CC for the LW group presents an increase over the controls, the CT and LW NMI distributions are very similar. For the GM-FD pair, there is an increase in NMI in the LW group, with a significant difference to the CT group. This increase can also be seen when compared with the MH group, although the difference is not significant. 

The CG-Causality parameter distribution for the two muscle pairs with significant differences between the groups is shown in Figure 5. As this parameter also presents directionality, one of the directionalities (e.g., TA → ED) is shown in black whiskers, while the opposite (e.g., TA ← ED) is shown in grey. The CG-Conditional values for the MH group show a clear reduction of the muscle interaction between TA-ED and GM-FD in both directions, in line with previous results. A slight increase can be seen in the LW group when compared to CT, although in this parameter it is not as pronounced as in the NMI. All the population groups presented a statistically significant foot-to-leg directionality (red background) of information transfer, except for the TA-ED interaction in the LW group.

Figure 6 represents the distribution of the TE muscle pairs with significant differences between groups. All the muscle pairs present results consistent with those of the previous metrics: increased muscle interaction for the LW group and reduced muscle interaction in the MH group when comparing both to the CT group. The decrease of the MH group can be especially seen in the TA → GM muscle pair direction and is even greater for the TA-ED muscle pair, with statistically significant differences between MH and the other two groups (CT and MH) in both directions. Increased muscle interaction for the LW group appears in the GM-FD pair in both directions as compared to the CT group, as well as in the ED → FD interaction. In the GM → ED direction, the increase in the LW group stands out from the other two population groups. In the directionality results, all the groups present significant TA → GM information transfer. TA-ED shows results consistent with the CG-Conditional, while the CT and MH groups present significant foot-to-leg directionality. The GM-FD and GM-ED pairs only show statistically significant directionality for the CT group, unlike the results obtained in the CG-Conditional, where the three population groups presented significant FD → GM directionality. The ED-FD pair did not indicate any predominant muscle directionality in any group.

To summarize the results obtained in this study, Figure 7 gives a visual representation of the differences in the muscle network observed from the CC, NMI, TE, and CG-Causality parameters between the three population groups (CT-LW, CT-MH, and LW-MH). Each line connects two muscles, indicating the muscle interaction between that pair. Only the muscle interactions with statistically significant differences between groups are displayed. The color represents the value of the parameter for that muscle interaction. A line that is more intensely red indicates stronger connectivity between that muscle pair. The arrow in the parameters with directionality characterizes the net direction of that muscle pair. The bidirectional arrows represent muscle interactions without a statistically significant directionality. 

The TA-GM interaction shows differences between groups in the CC and TE parameters. Both present reduced muscle connectivity in the MH group compared to CT in the CC parameter and to LW in the TE parameter. The directionality from the TE parameter of that muscle couple indicates a transfer from TA to GM. The results from the TA-ED pair are the most consistent of them all, with differences between groups across all the parameters and with identical tendencies. The LW group presents an increase in this muscle interaction compared to CT, while the MH group displays the opposite behavior with reduced connectivity of that muscle couple compared to CT and LW groups. The directionality in this pair is from ED to TA in the CT and MH groups, while the LW group does not present such a well-defined directionality. 

The GM-ED muscle pair only presents differences in the TE parameter, with a significant increase in muscle connectivity for the LW group over the other two population groups. The NMI and TE parameters give identical results for the GM-FD interaction, an increase in muscle connectivity in the LW group compared to the CT group. While the CG-Causality parameter also presents this increase for the LW group to a lesser extent, this parameter also gives a decrease in the connectivity for the MH group in comparison with the other two.

Differences between the groups for the ED-FD pair were only found in the TE parameter, indicating an increase in the connectivity between this pair for the LW group compared to the CT group. In short, the results show consistency between the different information theory metrics, with the LW group showing an overall increase in connectivity between muscle pairs compared to the CT group and a decrease in muscle interactions for the MH group compared to the CT and LW groups. TE is the parameter with the largest number of differences between groups in multiple muscle pairs and can identify changes in the LW group in comparison to CT, which could be used as a valuable tool for early DPN detection.

## 4. Discussion

This study assessed intermuscular coupling in healthy subjects, diabetics with a low risk of DPN, and diabetics with a moderate/high risk of contracting this disease to evaluate network connectivity disorders associated with diabetes and neuropathy. Previous research has reported alterations in muscle activation patterns of leg and foot muscles in DPN patients compared to healthy volunteers [13,15,16,20]. The traditional sEMG parameters such as root mean square (RMS), median frequency (MDF), discrete wavelet transform (DWT) and sample entropy (SAMPEN) have also been studied in the context of DPN [21,32,33]. While traditional parameters focus on individual muscle characteristics, in contrast, our study presents a novel approach by evaluating changes in the muscle network rather than focusing solely on individual muscles. This approach helps to uncover how alterations in muscle coupling and coordination contribute to the overall muscle network changes in DPN. Intermuscular coupling has also been analyzed in other contexts, including post-stroke patients during gait, muscle coordination with exoskeletons, muscle fatigue conditions, and functional interactions in healthy subjects [22,23,25,34]. To the best of our knowledge, the impact of diabetes and DPN on muscle network connectivity has not been documented in the literature.

The four information theory metrics analyzed in the study (CC, NMI, CG-Causality, and TE) indicated that TE presented the highest number of statistical differences between groups in all muscle pairs (except TA-FD) providing directionality information, unlike CC and NMI. This corroborates the presence of differences in non-linear relationships in muscle coupling between groups that are indetectable by GC [18]. This finding was also reported in a previous study in which multivariate sample entropy, a non-linear theory metric similar to TE, was more sensitive to changes in muscle coupling than CG-Causality [25]. 

Of the six muscle pairs analyzed, only the TA-FD pair did not indicate differences between any group or parameter. The TA-ED muscle pair yielded the most consistent results across all the parameters analyzed, showing statistically significant differences between the moderate/high risk (MH) group and the other two groups (control (CT) and low risk (LW)), with a decrease in muscle coupling and information transfer in the presence of DPN. However, this muscle pair did not differentiate LW from the CT group, which would enable early detection of the pathology. Previous studies have found alterations in TA muscle activation patterns in diabetic patients, particularly in those with DPN, who exhibit reduced MU recruitment and slower contractile properties [12]. Differences in TA muscle activation between diabetics with and without DPN have been observed in gait, though not consistently between controls and DPN patients [13,15,16]. This suggests that the muscles responsible for dorsiflexion exhibit lower connectivity, potentially related to DPN severity and characterized by axonal degeneration and muscle fiber loss [6]. TE and CG-Causality showed consistent directionality results for this muscle pair, with significant information transfer from ED to TA in the CT and MH groups but not in the LW group, indicating possible compensatory neuromuscular regulation in the early stages of DPN [13]. This reduced information transfer between the TA, GM, gastrocnemius lateralis, and soleus ankle muscles has previously been reported in post-stroke patients during the double support phase of gait [23].

The GM-FD muscle pair also showed consistent results across the parameters. The general trend indicated higher muscle coupling in the LW group than in the CT group and reduced coupling in the MH group. CG-Causality was particularly sensitive to the lower muscle coupling in the MH group, while NMI and TE were more selective in detecting increased intermuscular coupling in the LW group, which could be a promising biomarker for early DPN detection. The directionality results for this muscle pair showed a significant information transfer from FD to GM in the CT group, but this did not appear in the diabetic cohort (LW and MH).

The fact that the TA-ED and GM-FD muscle pairs showed the best discrimination results could be due to anatomical reasons. The muscles in each pair are innervated by the same nerves, which further support their coordination. The TA and ED muscles are both innervated by the deep fibular (peroneal) nerve [35], which ensures that signals for muscle activation are transmitted simultaneously, facilitating synchronous movement patterns. The GM and FD muscles are innervated by the medial plantar nerve branch of the posterior tibial nerve [35], another shared neural pathway that promotes coordinated activation. The foot-to-leg directionality could be due to the intrinsic biomechanics of the dynamic exercise. The muscle recruitment for the movement originates in the toes, followed by the rest of the foot, which then engages in dorsiflexion or plantar flexion at the ankle.

CC, NMI, CG-Causality, and TE revealed differences in the neuromuscular coupling mechanisms in diabetic DPN patients. The LW group showed increased muscle network connectivity over CT, while MH was lower than CT. Apart from what was found in the TA-ED and GM-FD pairs, there are other muscle pairs that corroborate this trend. The reduction in the MH group is also present in the TA-GM muscle pairs in the CC and TE parameters, while the increase in muscle connectivity of the LW group is also present in other muscle pairs, such as the GM-ED and ED-FD in the TE parameter. The decrease in the MH group may be due to multiple mechanisms such as joint stiffness, lack of coordination from loss of proprioception, and primarily the intrinsic loss of MU, leading to reduced information transfer in the muscle network than in people without DPN [12,36]. Conversely, the increase in the LW group could have been due to the compensatory mechanisms for sensorimotor deficits. Although this group does suffer a certain loss of MU, it has a smaller effect, and the reinnervation following denervation may contribute to greater information transfer within the muscle network than in healthy individuals [6,36]. Identifying these changes in the LW group is fundamental for obtaining biomarkers for early detection of DPN and targeting preventive interventions. 

The CT group showed significant foot-to-leg information transfer, although not as clearly as in the LW group. This lack of a predominant directionality could result from compensatory neuromuscular regulation strategies, as the LW group may be in the early stages of DPN, experiencing common ankle stiffness and MU loss in diabetic individuals. The TA muscle could thus compensate by increasing and balancing information transfer in both directions. In contrast, MH patients are unable to develop compensatory strategies due to their sensorimotor deficits, leading to weaker muscle coupling [13].

These findings suggest the potential use of these metrics to provide objective feedback on the therapy effectiveness of motor control and muscle recruitment strategies to reveal DPN-related neuromuscular mechanisms. It should be noted that peripheral arterial disease (PAD), a common complication of diabetes, could significantly impact muscle connectivity [37,38]. As we did not include any subjects with advanced PAD, future studies should focus on the effects of PAD on these parameters in the more advanced stages. As the present study’s main limitation was the small sample size, future research with larger samples and an analysis of more muscles bilaterally could gain further insights into motor strategies. 

## 5. Conclusions

The presence of diabetes and DPN deeply affects the intermuscular coupling mechanisms, manifesting differently depending on the degree of severity of the disease. An increase in the information transfer and muscle connectivity in the LW group in comparison with CT was observed, while the MH group presented a significant decrease in comparison with CT. 

Of the four parameters analyzed, TE outperformed CC, NMI, and CG-Causality in detecting coupling alterations in DPN patients. This parameter provided statistically significant differences in both the quantity and directionality of information transfer between the groups of most of the muscle pairs. 

The TA-ED pair showed the most consistent performance across all four parameters. As the GM-FD and GM-ED couples differentiated the LW group, they could be potential biomarkers for early DPN detection.

These findings have the potential to reveal the crucial neuromuscular mechanisms for clinical practice, help to develop appropriate rehabilitation strategies, and serve as biomarkers for monitoring muscle synergy evolution. Using TE to assess muscle network connectivity can provide deeper insights into the complexities of neuromuscular coupling in DPN patients and facilitate more targeted and effective therapeutic interventions.

## Figures and Tables

**Table 1:** ANOVA test *p*-values for a general comparison between groups (CT, LW, and MH) for CC, NMI, CG-Causality, and TE computed across the six muscle pairs. The arrow in the CG-Causality and TE parameters indicates directionality. The statistically significant *p* values (*p* < 0.05) are shown in bold with (*).

Pair	CC	NMI	CG-Causality →	CG-Causality ←	TE →	TE ←
TA-GM	0.19	0.40	0.56	0.85	0.13	0.22
TA-ED	0.00 *	0.00 *	0.01 *	0.00 *	0.00 *	0.00 *
TA-FD	0.99	0.25	0.46	0.77	0.34	0.59
GM-ED	0.60	0.59	0.08	0.09	0.00 *	0.16
GM-FD	0.55	0.01 *	0.06	0.10	0.02 *	0.07
ED-FD	0.76	0.27	0.25	0.20	0.51	0.15

**Table 2:** *p*-values of the LSD test comparing the CT, LW, and MH populations for CC and NMI computed across the six muscle pairs. Figures in bold with (*) are the statistically significant *p*-values (*p* < 0.05).

	CC			NMI		
Pair	CT-LW	CT-MH	LW-MH	CT-LW	CT-MH	LW-MH
TA-GM	0.07	0.01 *	0.83	0.17	0.82	0.61
TA-ED	0.11	0.04 *	0.00 *	0.71	0.00 *	0.00 *
TA-FD	0.93	0.20	0.29	0.33	0.20	0.92
GM-ED	0.41	0.71	0.31	0.40	0.30	0.12
GM-FD	0.18	0.75	0.15	0.00 *	0.43	0.07
ED-FD	0.76	0.22	0.23	0.37	0.10	0.66

**Table 3:** LSD test *p*-values comparing the CT, LW, and MH populations of CG-Causality and TE. Figures in bold with (*) are the statistically significant *p*-values (*p* < 0.05).

	CC			NMI		
Pair	CT-LW	CT-MH	LW-MH	CT-LW	CT-MH	LW-MH
TA → GM	0.08	0.12	0.70	0.11	0.54	0.05 *
TA ← GM	0.32	0.11	0.75	0.57	0.30	0.20
TA → ED	0.61	0.01 *	0.01 *	0.67	0.00 *	0.00 *
TA ← ED	0.35	0.00 *	0.00 *	0.63	0.00 *	0.05 *
TA → FD	0.86	0.60	0.81	0.20	0.80	0.18
TA ← FD	0.89	0.39	0.59	0.90	0.39	0.44
GM → ED	0.65	0.07	0.34	0.01 *	0.19	0.00 *
GM ← ED	0.62	0.09	0.40	0.59	0.15	0.11
GM → FD	0.74	0.04 *	0.07	0.01 *	0.64	0.07
GM ← FD	0.55	0.05 *	0.04 *	0.03 *	0.65	0.10
ED → FD	0.87	0.15	0.22	0.57	0.36	0.23
ED ← FD	0.10	0.28	0.50	0.04 *	0.94	0.06

## Data Availability

The datasets presented in this article are not readily available because the data are part of an ongoing study. Requests to access the datasets should be directed to the corresponding author.
